# A novel approach to research engagement: Developing a targeted theory of change with Black and African-American stakeholders

**DOI:** 10.1017/cts.2024.610

**Published:** 2024-10-07

**Authors:** Helen Hemley, Juliana M. Ison, Marissa Reynolds, Tiffany Pham, Jonathan D. Jackson

**Affiliations:** 1 CARE Research Center, Massachusetts General Hospital, Boston, MA, USA; 2 CRESCENT Advising, LLC, Belmont, MA, USA; 3 University of California Santa Barbara, Santa Barbara, CA, USA; 4 Harvard Medical School, Boston, MA, USA

**Keywords:** Theory of change, community engagement, clinical research, outcome map, stakeholder group

## Abstract

**Purpose:**

Community inclusion in research may increase the quality and relevance of research, but doing so in an equitable way is complex. Novel approaches used to build engagement with historically marginalized communities in other sectors may have relevance in the clinical research sector.

**Method:**

To address long-standing gaps and challenges, a stakeholder group was convened to develop a theory of change (ToC), a structured method for obtaining input from stakeholders to enhance the design, conduct, and dissemination of research. The stakeholder group, comprised of Black residents within a metropolitan area, followed a structured monthly meeting schedule for 12 months to produce an outcome map, a model that formally defines aspects of research and engagement for this community.

**Results:**

Stakeholders reported significant improvements in trust in and engagement with research over the 12-month period, but not changes in health empowerment (individual, organizational, or community level). Through this convening process, a ToC and outcome map were developed with the focus of building bidirectional relationships between groups identifying as Black, Indigenous, and People of Color (BIPOC) and researchers in Boston, MA. Additionally, the group developed a community ownership model and guidelines for researchers to adhere to when utilizing the ToC and outcome map with BIPOC communities.

**Conclusion:**

Co-ownership of models to develop bidirectional relationships between researchers and community members, such as the ToC and outcome map, may advance and further the value and reach of community-based participatory research while increasing levels of trust and engagement in research.

## Introduction

Patients from historically marginalized and minoritized (HMM) populations are disproportionately burdened by a wide spectrum of diseases and disorders yet most clinical trials lack representative enrollment based on this risk profile [1]. Community involvement may increase the quality and relevance of research, yet enhancing community leadership in research design is a central challenge in advancing equitable community-based research [2]. Despite increasing awareness of inequities for HMMs in clinical research, studies continue to engage and recruit HMM groups as a monolith, despite readily evident historical, cultural, and geographic differences between communities and individuals [3]. Without appropriate training or experience, attempts to facilitate community engagement are often ineffective, burdensome, and leave stakeholders feeling disenfranchized [4].

Few clinical studies have sufficient personnel, training, or resources dedicated to engaging prospective participants about research. Consequently, researchers without prior experience have limited options for HMM engagement. Additionally, infrastructure at many academic health centers is not well aligned to support community engagement [2]. Significant gaps still exist in the methods used to engage communities in research, and the process is often resource-intensive, ad hoc, and time-consuming for individual researchers and communities alike [2,5,6]. Thus, researchers engaged in community-based participatory research (CBPR) often report difficulties around model sustainability, community buy-in, and reinforcing existing social hierarchies. To address the need for eliciting meaningful research engagement across research studies and disease areas, we convened a targeted stakeholder group (TSG) to develop a theory of change (ToC), providing a structured method to obtain input from stakeholders that enhances general research design, conduct, and dissemination [7]. Differing from a community advisory board (CAB), this group was convened to focus on a specific community’s relationship with research overall – Black and African Americans in Boston, MA. Further distinctions include the group’s focus on building models to resolve long-term issues around research engagement and bidirectional benefit, TSG members having leadership roles in the decision-making process across and after the project period to increase empowerment and interest in research on the individual level, as well as the amount of compensation provided to TSG members to be commensurate with their expertise.

Currently, the burden of HMM clinical trial participation is on communities disconnected from health systems, individual research teams without sufficient resources to reach these communities, and disempowered HMM individuals required to navigate access gaps on their own [4]. This ad hoc response to HMM participation incentivizes study teams to conflate research engagement with recruitment. Without a clear distinction between these activities, HMM communities may be led into or away from research studies without empowerment during vital decisional stages, a foundation of patient-centered approaches to research. Introducing a ToC is an innovation that will advance the importance of centering community needs in research design, as it is an iterative process across either a study timeline or to enhance community–research relationships overall, thus providing clear steps from potential participant engagement to recruitment to retention that are not only based in CBPR theory but also responsive to community leaders’ edits and expanded group feedback as the ToC process unfolds.

## Materials and methods

A guided approach to community engagement, the TSG framework allows stakeholders to be expert consultants in design processes, rather than convened for ad hoc review after major decision-making stages are complete. TSG development relies on a public health model [8], modified for clinical research spaces, to identify stakeholders across diverse experiential backgrounds, minimizing the phenomena of tokenization and maximizing opportunities to hear from multiple community touchpoints.

### Composition of targeted stakeholder group

**Figure 1.**
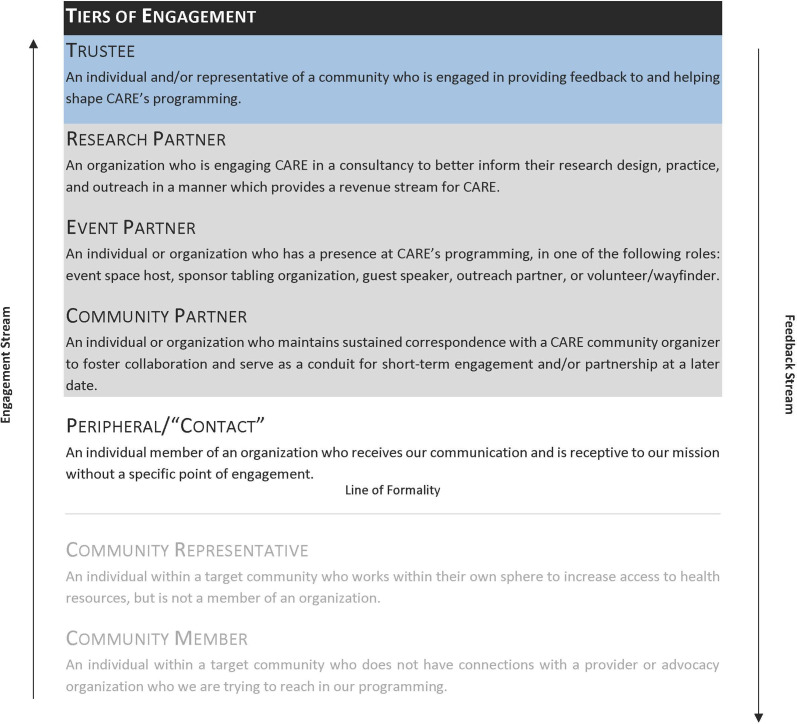
Stakeholder engagement ladder.

TSG makeup is modeled using a modified stakeholder engagement structure [8] for determining potential partnerships and a stakeholder engagement ladder, both developed for clinical research spaces. In the stakeholder engagement structure, a review process of potential stakeholders is initiated to ensure bidirectional partnerships with community members are established based on individual expertise and background, ensuring the TSG approaches presented problems from multiple angles. TSG members are compensated at rates based on the local mean for consultancy time, ensuring appropriate value for their expertise. This level of expertise and partnership is further validated by the stakeholder engagement ladder (Fig. 1), a tiered engagement structure which categorizes and tracks developing community engagement relationships, determining which community leaders have expressed a desire to serve at the level of research partner or trustee for their representative community.

Utilizing both the stakeholder engagement ladder and the modified stakeholder engagement structure, leaders in Boston who self-identified as Black and/or African American were approached by the study team from the following sectors: Public Health; LGBTQ + Advocacy, Community Health; Hospitality; Nursing; Higher Education; Public Education; Faith & Ministry; Housing; and Elder Advocacy. Leaders were assessed by the study team on their stakeholder engagement ladder at two time points: at consent and the project’s conclusion. Out of the 11 leaders originally approached to participate in the group, only one did not respond and all respondents agreed to participate. Upon the first convening, the group identified two additional sectors for intersectional representation on the TSG, Clinical Research and Youth Advocacy. The participants first approached to serve in these capacities agreed and were present at all TSG convenings from Meeting 2 onwards.

### Project deliverables

The primary qualitative outcomes of this project are the ToC (Supplemental Material 1), outcome map, and guidebook (Supplemental Material 2), developed by the stakeholder group. Each of these deliverables was created to enhance empowerment, education, and engagement for Black, Indigenous, and People of Color (BIPOC) communities when working with research systems. These tenets were chosen by the stakeholder group and are reflected in each document. Qualitative themes and analysis took place during each of the nine stakeholder group meetings, the transcribed content of which resulted in the community-developed materials. Given the collaborative nature of the meetings, this process was iterative, with the TSG members reviewing, editing, and approving multiple versions of each document. Stakeholders reported feeling proud of the final versions of said materials and are looking forward to sharing them with the broader community for feedback.

The primary quantitative outcomes of this project are the results of three surveys across three distinct time points. Three surveys measuring trust, engagement, and community empowerment were collected via REDCap, an online data collection platform, across three time points – Meeting 1, Meeting 5, and Meeting 9 – to assess any changes in attitudes in trust, empowerment, and engagement in research across the project.

#### Trust in Medical Researchers Scale (TIMRS)

The Trust in Medical Researchers Scale (TIMRS) [9] is a validated 12-item self-report scale and uses a 5-point Likert scale (“strongly agree” to “strongly disagree”) to measure responses, with an additional option for “I don’t know.” It is comprised of two subscales, participant deception and researcher honesty.

#### Patient Engagement in Research Scale (PEIRS)

The Patient Engagement in Research Scale (PEIRS) [10] is a validated 37-item self-report scale and uses a 5-point Likert scale (“strongly agree” to “strongly disagree”) to measure responses, with additional options for “I don’t know” and “not applicable.” Example statements from the instrument include “I was interested in the issue(s) being researched in the project;” and “I was an equal partner in the research project team.”

#### Health Education and Community Empowerment Survey (HECES)

The Health Education and Community Empowerment Survey (HECES) [11] is a validated 12-item self-report scale and uses a 5-point Likert scale (“strongly agree” to “strongly disagree”) to measure responses, with additional options for “I don’t know” and “not applicable.” The survey is comprised of three subscales: individual level change, organizational level change, and community level change.

### Theory of change

While TSGs can be convened for a myriad of purposes to achieve specific goals, this project convened a TSG to develop a ToC. A ToC is a work-flowed, stakeholder-based process to ensure that developed solutions serve inclusive populations within an identified community [12]. A ToC provides a seamless, coalitional approach that is robust to change over time, allowing partners and stakeholders to define and then map strategies continually and update their relevance to serve identified outcomes and emergent needs. It is the intent of the process of building a ToC to help answer important questions about the nature of the proposed problem and engage stakeholders in the process of building a solution that derives a clarity of purpose, an understanding of assumptions and a strengthened resolve in their expertise in defining a process-led solution for a proposed problem.

Building a ToC helps answer important questions about the nature of proposed problems and engages stakeholders in the process of building solutions that derive a clarity of purpose, understanding of assumptions, and strengthened resolve to define process-led solutions. ToCs are a well-validated framework and, while adaptable in terms of problem scope, project length, and group size, have accessible and clear guidelines for use for new facilitators, making them an approachable and adaptable tool for not only participants but study teams [13]. ToCs have advanced equitable outcomes across multiple sectors, but this approach was not yet leveraged in clinical research settings. Crucially, ToCs encourage the development of concrete, patient-centered mechanisms to advance clinical research activities via specific tools such as an outcome map, empowering communities to define the distinction between research engagement and recruitment on their own terms.

### Outcome map

An outcome map is a methodology that relies on outcomes as behavioral change for both institutions and individuals [13]. Outcome maps allow TSGs to measure impact across multiple engagement strategies and entry points. Additionally, they serve as actionable diagrams that demonstrate and visualize how the desired outcomes of the ToC can be facilitated at individual, community, or organizational levels. Clinical trial teams, limited in resources to advance equitable engagement and recruitment, often look for a single solution to recruit HMMs to research trials. Working backward from the stated goal, outcome maps create an opportunity for stakeholders to identify needed changes and then determine which interventions will best achieve long-term goals. Because outcome maps are developed to ensure that all elements of long-term goals defined by the ToC are fully considered [12,13], introducing this tool into clinical research spaces is itself a significant innovation over short-term, myopic solutions.

### TSG meetings

**Figure 2.**
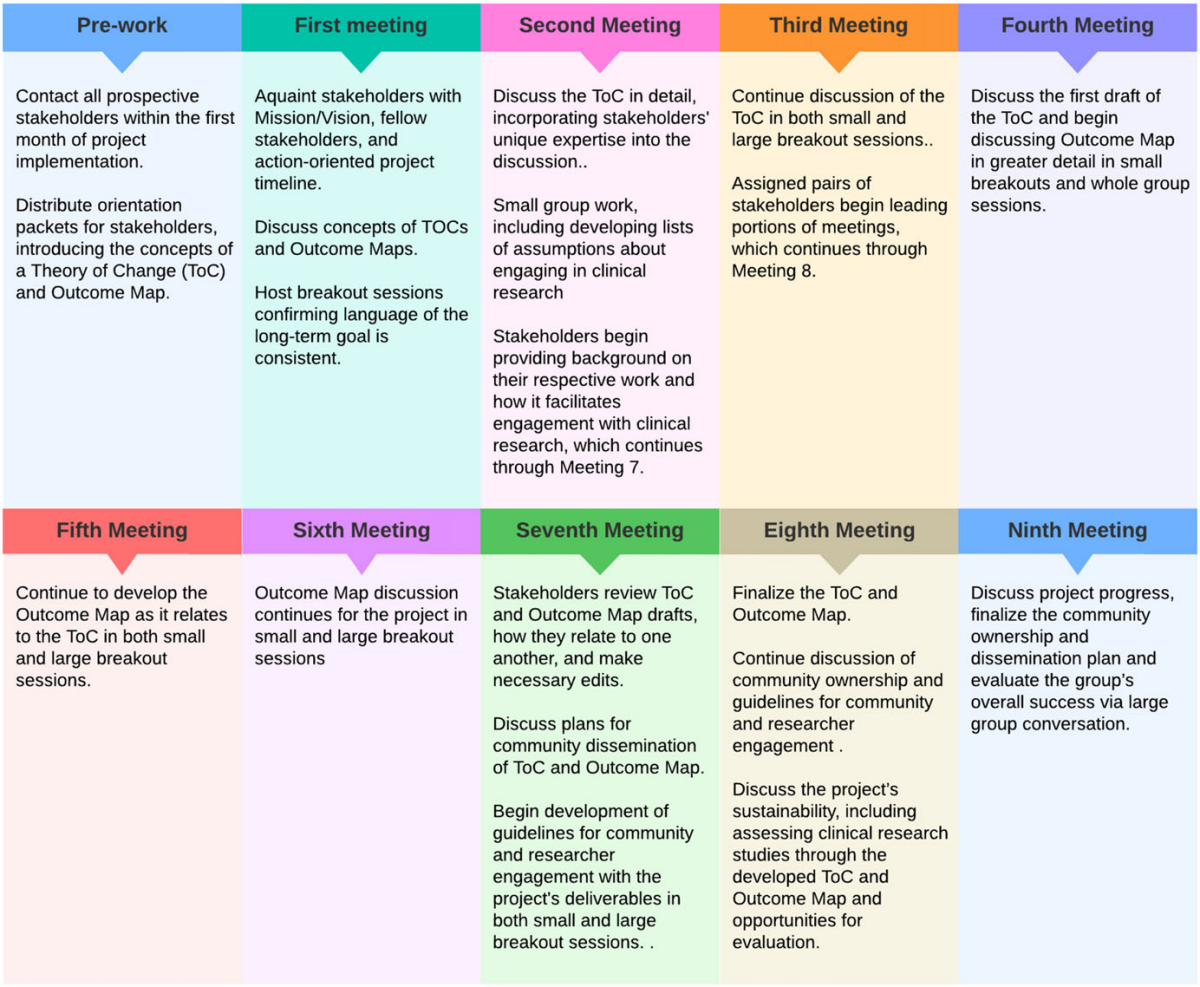
Targeted stakeholder group meeting workflow.

The ToC development process requires once-a-month TSG convening across a nine-month period. The planning and TSG recruitment process is dependent on the level of community entrenchment the research team has, generally taking between one and three months. Stakeholders are expected to lead portions of each meeting, evaluate engagement practices between meetings, and present their perspective on community engagement, empowerment, and education within and outside of clinical research systems. Each TSG meeting is two hours in length. To prepare stakeholders, the project lead provided an orientation packet and digital access questionnaire, determining whether additional accommodations were required for full project participation. Due to the COVID-19 pandemic, all TSG convenings took place virtually on a secure Zoom account platform. Funds were provided by PCORI to provide connectivity devices for participants, if needed, to ensure digital access was not a barrier to participation. The project’s meeting structure (Fig. 2) was developed using a Community Partnership Review Process [8]; the Stakeholder Engagement Ladder was utilized to understand community partner relationships.

The estimated cost for a nine-month ToC development process via a TSG is $39,000, including a HIPPAA-protected virtual meeting subscription, diagramming service subscription, and stakeholder compensation. Stakeholders were compensated at a rate of $175 per hour, totaling $3,150 for each group member. For this implementation, payments were divided into three time points which were tied to survey completion to determine the efficacy of our approach. There may be additional costs for digital devices, participation support (e.g., transportation and childcare), and interpretation.

## Results

### Qualitative analysis

As a result of the nine TSG meetings, qualitative key findings included the belief across the stakeholder group that mistrust of research systems is influenced by but not primarily driven by historic atrocities like the Tuskegee Syphilis study or continued abuses against Henrietta Lacks and her family [14,15]. Rather, current interactions with healthcare and research systems that perpetuate inequities in health result in mistrust of these systems [16]. Stakeholders noted discrimination of BIPOC communities by healthcare and research workers, alike, and how the public is often unable to distinguish between these two groups when they work for the same institution. Furthermore, research education and opportunities were noted as not being offered to all patients at the same level, which can be perceived as a systems-level assumption that BIPOC will not want to be included in research unless diversity is overtly stated due to historic mistrust. Stakeholders noted that it is not the community’s responsibility to gain research literacy and improve research equity, rather institutions must invest in systemic change both internally and within the community to ensure there is equity in research representation as well as acknowledgment of the expertise that resides within BIPOC communities. Quotes from stakeholders relating to trust and engagement with research to date include:

- Researchers aren’t from Boston and don’t know the history of their own institutions and the communities they’re trying to engage. They don’t understand why they’re not welcome.
- What are people going to do with the data? We have to be at the table asking the questions, not just being used as guinea pigs, because that has been the reality.
- Researchers must remember that if they want to get people involved in research, asking them to get involved in research is not the first question. It is important to meet people where they are, understand why they are there, and begin to talk about the things that interest them and their community.

The 12 stakeholders engaged in this project each reported overall positive interaction and impact from participating. Using the stakeholder engagement ladder as a measure of each stakeholder’s increased engagement with clinical research, the study team assessed the TSG members’ placement on the ladder at consent and study closeout. Three group members were categorized as community members prior to involvement in the TSG, out of which all three had their positions elevated by three levels to community partners, evidenced by continued contact with the study team and engagement with the project by writing letters of support for project continuation and requesting their names to be associated with the project results. One stakeholder member was defined at the level of community representative and changed their standing by five levels to trustee, engaging in the previously mentioned metrics and additionally finding a career in clinical research engagement. One TSG member entered the project at the level of “Peripheral”/Contact and at the convenings’ end increased by three levels to research partner, actively looking to apply to additional funding sources for the project’s sustainment. Three TSG members entered the project at the level of community partner and rose by two levels to research partner, engaging in multiple events post-award to promote the project’s outputs and findings. Two TSG members entered at the level of event partner and stayed at this level at the conclusion of the convenings. Two TSG members entered the project at the level of trustee and stayed at this level at the project’s conclusion.

Each stakeholder has verbally committed to continuing this work, either by continuing as a stakeholder or providing ad hoc guidance on how to engage with the sector they represented to sustain the project. Multiple stakeholders have presented on the findings and their experiences to clinical researchers, community members, health systems professionals, and state and federal government representatives. Additionally, each member of the TSG requested to have their name associated with the project when presented with the opportunity at consent for each survey time point. This level of project buy-in demonstrates that using this design to engage stakeholders at the leadership and study design level can increase study engagement and retention, while also producing an increase in trust, engagement, and perceived honesty, especially for persons who were previously unfamiliar with or adjacent to clinical research, as evidenced by the increased engagement on the stakeholder engagement ladder of persons previously unaware of the study team’s work. Given that these indicators of engagement – continued and consistent opportunities for communication, public leadership opportunities and recognition, regular evaluation of success – are what TSG members included as metrics of success in the ToC and outcome map, as well as their statement of community ownership of materials, the TSG is directly responsible for these measures of reliability in the group’s outcome toward project engagement and retention. Quotes from stakeholders relating to building positive relationships with research include:

- We want to make sure that by the time we get there…we’re not just showing up for the ask. We’re showing up because we’ve built a relationship, we’ve built trust, we’ve build a level of understanding and clarity around what it is that we’re doing, why it makes sense for people based on what they perceive as having value, not just what researchers perceive as having value in terms of more successful community engagement research.
- An important goal for research–community partnerships is to leave the community better than researchers found it. How can the research be a value added rather than a transaction? There are likely other things that are important to the community outside of just the research team’s research question, so it is vital the research team incorporates ideas of how to give back beyond simply the scope of just the research question.
- Sustainable change and engagement has to happen at a system/institutional level. If it’s just individual researchers building relationships with individual groups around narrow topics then that is not going to create lasting change and engagement we want to see. Institutions have to play a role in building long-term relationships and providing capital for investments. If we want to engage communities we need to acknowledge and support people’s time. That has to come from institutions because researchers don’t have the money for that.

**Figure 3.**
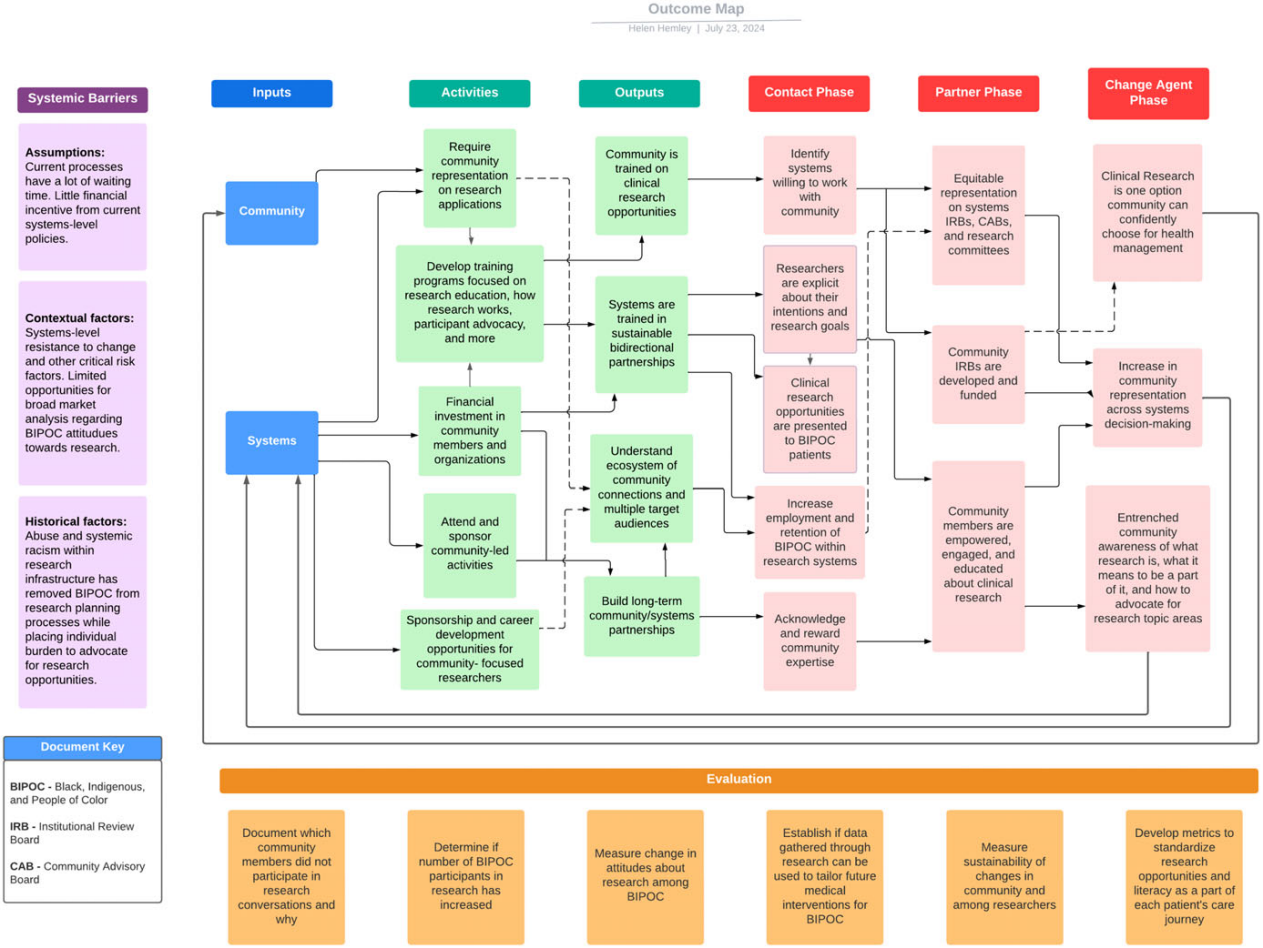
Stakeholder-developed outcome map.

These key findings are demonstrated in plain language that is accessible to researchers, community members, clinicians, and institutions in the stakeholder-created ToC (Supplemental Material 1), outcome map (Fig. 3), and guidebook (Supplemental Material 2). Stakeholders stressed the need for evaluation of these processes outside of the number of persons attending engagement events and have therefore created cyclical tools to ensure engaging with these materials is not a one-time event but a process to evaluate research equity and community empowerment for both researchers and BIPOC communities on a continual basis. Through the Toc (Supplemental Material 1), the stakeholder group identified the key areas of engagement, education, and empowerment as essential to building transformative change in research spaces for BIPOC and to build bidirectional relationships between HMM communities:

*Our Theory of Change model addresses barriers to research via three channels: Empowerment, Engagement, and Education. We define empowerment here as a patient’s ability to freely ask questions, feel fully confident to advocate on their own behalf in research matters, understand how a study will affect their health and/or insurance, and know what is required of them and/or their community if they participate. We define engagement as any interaction with research outside of recruitment, such as participating in research design, attending informational sessions and giving feedback, or having a chance to learn more about research outside of clinical settings. The burden of effort in engagement activities should fall on researchers and institutions. They must be willing to travel to the community to ensure these opportunities are accessible. Additionally, they must partner with people from the community who understand the dynamics of race relations in Boston. Finally, we define education in three parts: First, as the training of research staff and institutions in current barriers and health concerns facing BIPOC communities in Boston and eliminating discrimination in the research process. Second, as the direct education of the community by research staff in research and its benefits for present and future generations. Third, as institutions building long-term relationships with the community and providing capital to compensate community members for their time spent on these partnerships.*

The outcome map (Fig. 3) developed by the stakeholder group displays a clear journey for researchers and community members alike to actualize the concepts discussed in the ToC through clear action, beginning with understanding of systemic barriers for research participation for BIPOC communities [5,6], community members and healthcare/research systems as inputs for success of the work, activities for inputs to complete, outputs as a result of said activities, and a process for systems to move from being community contacts, to partners, to change agents. This iterative process is meant to be continuously evaluated via community-defined metrics, demonstrated as such across the outcome map and, as such, will never exist in a final form as it will always be responsive and elastic to introduce emerging community needs for long-term engagement with clinical research systems. From the stakeholder-developed Guidebook (Supplemental Material 2):

*Before research even happens, there needs to be a conversation with the community first. That is the intent of this outcome map – to start an ongoing, productive, and bidirectional relationship between communities identifying as Black, Indigenous and People of Color (BIPOC) and clinical research systems. This outcome map was not developed to recruit BIPOC persons for a single study or to ask for a collaboration with clear start and end dates. It is meant to start an ongoing process where both BIPOC communities in Boston, MA and clinical research systems have multiple entry points to reviewing whether the structures created together answer the following questions:*

*A) Are we changing the proportion of BIPOC individuals who are participating in research?*

*B) Are we building enough data to be used as a foundation, so that medical and scientific communities can tailor interventions for the high-risk populations we’re discussing in the same way that privileged populations are?*

*C) Are attitudes about research among BIPOC changing to become more accepting of research as a treatment option?*

*It is the hope that by using the outcome map as a guide for building this infrastructure that both BIPOC and researchers will benefit from expanding scientific knowledge of disease areas and that BIPOC communities will directly benefit from this research by engaging with clinical research on their own terms through a variety of entry points outside of formal recruitment. However, utilizing the outcome map shouldn’t be the lead consideration when building towards this goal. It should be what research systems and communities alike use as a way to frame the results of the successful process of moving through the theory of change.*

Finally, the stakeholder developed guidebook (Supplemental Material 2) for researcher utilization of the ToC and outcome map demonstrate a clear path for community ownership of research materials developed by and for BIPOC communities:

*This outcome map was developed by 12 stakeholders from the Greater Boston Area who self-identify as being from BIPOC communities. As a result, this map should not yet be considered a final version as it has not received broader community input. As the research team begins the process of gathering greater community feedback to finalize this map, this stakeholder group asks persons interacting with the outcome map to contact the group through the research team to receive guidance on appropriately disseminating and replicating this work. It is fully the intent of the stakeholder group to allow other iterations of this work to grow and flourish, but as this work is in the early stages, we must acknowledge the potential ease of having this outcome map used for unintended purposes and will act as gatekeepers for the immediate future.*

### Quantitative analysis

#### Data analytic strategy

Means, medians, and standard deviations were calculated for each of the three scales (TIMRS, PEIRS, and HECES). Researchers then performed repeated measures ANOVAs and Bonferroni post hoc corrections for each of the scales to measure longitudinal changes across survey time points (Table 1). All analyses were conducted using version 3.6.1 of the statistical analysis software R.

**Table 1.** Summary descriptives for stakeholder surveys

	Time point 1		Time point 2		Time point 3	
	*M*	*SD*	*M*	*SD*	*M*	*SD*
Patient Engagement (PEIRS)	85.58	46.33	41.95	10.54	37.90	8.71
Researcher Trust (TIMRS)	35.08	6.68	37.85	7.32	34.46	7.33
*TIMRS - Participant Deception*	18.17	3.86	19.23	4.62	16.85	3.91
*TIMRS - Researcher Honesty*	16.92	3.23	18.62	3.48	17.62	4.01
Health Empowerment (HECES)	27.33	5.16	27.31	5.39	25.15	3.74
*HECES - Individual*	4.00	1.60	4.00	1.53	3.85	1.63
*HECES - Organizational*	13.25	3.62	12.46	3.95	11.23	2.35
*HECES - Community*	10.08	1.68	10.85	1.72	10.08	1.89

PEIRS = Patient Engagement in Research Scale; TIMRS = Trust in Medical Researchers Scale; HECES = Health Education and Community Empowerment survey.

#### Survey results

Summary descriptives for the three surveys are reported in Table 1. Data were analyzed for all stakeholders across the three time points. One stakeholder was missing a single time point for one of the collected scales. This time point, along with other missing data points (i.e., responses that were classified as “unsure” or “not applicable”) were replaced with imputed data by group item average. Aggregated sums were used in the analysis of each scale.

For PEIRS, a Bonferroni-corrected repeated measures ANOVA demonstrated a significant statistical difference between the patient engagement scores across survey time points (*F* = 9.192, *p* = 0.001) with a moderate effect of time on patient engagement scores (η^2^ = 0.19, 95% CI [0.00, 0.40]). An accompanying Bonferroni post hoc correction revealed significant valenced directional changes between survey time points one and three. Between time points one and three, a large effect size was found (*d* = −1.126; 95% CI [−1.810, −0.442]). Although a pairwise *t*-test with accompanying Bonferroni post hoc correction did not report a significant difference between time points one and two, a medium effect size was found (*d* = −0.784; 95% CI [−1.560, −0.007]).

Researchers collected data for TIMRS’ two subscales: participant deception and researcher honesty. Researchers found significant differences in both subscales, with *F* = 3.981 and *p* = 0.033 for participant deception and *F* = 4.535 and *p* = 0.022 for researcher honesty. The overall effect size for the participant deception subscale (η_p_^2^ = 0.266) was considered large. Although pairwise *t*-tests with accompanying Bonferroni post hoc corrections for participant deception did not report significant differences between any time points, a small effect size was found between time points 2 and 3 (*d* = −0.482, 95% CI [−0.923, −0.042]). In addition, the effect size for researcher honesty (η_p_^2^ = 0.292) was considered large. Pairwise *t*-tests with accompanying Bonferroni post hoc corrections for researcher honesty did not report significant differences between any time points, but a medium effect size was found between time points one and two (*d* = 0.525, 95% CI [0.096, 0.955]).

The HECES, composed of three subscales: individual level change, organizational level change, and community level change, was also collected. Repeated measures ANOVAs as well as Bonferroni post hoc corrections yielded no significant results for the individual level change and organizational level change subscales; the effect sizes were negligible for these measures. For the community-level change subscale, an overall large effect of time points on Community-level change scores was found (η_p_^2^ = 0.161), with a medium effect size between time points two and three (d = −0.751, 95% CI [−1.470, −0.032]).

## Discussion

**Figure 4.**
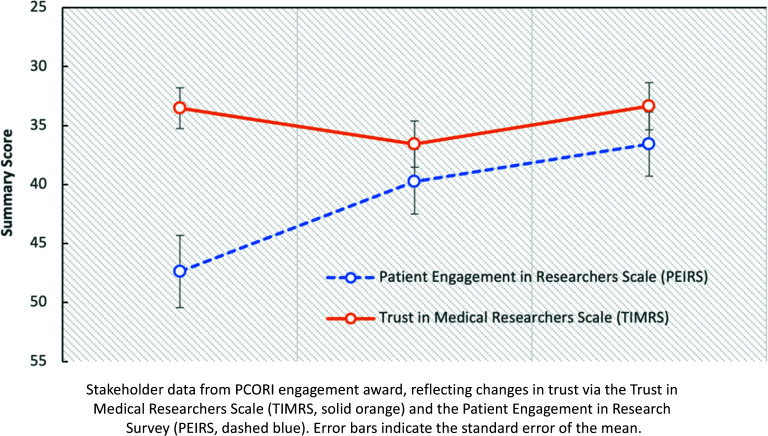
PEIRS [10] and TIMRS [9] survey results. Stakeholder data from Patient Centered Outcomes Research Institute (PCORI) engagement award, reflecting changes in trust via the Trust in Medical Researchers Scale (TIMRS, solid orange) and the Patient Engagement in Research Survey (PEIRS, dashed blue). Error bars indicate the standard error of the mean.

The community response to this effort demonstrates interest in clinical research participation via input and leadership in scientific design. Stakeholders remained engaged through the duration of TSG convening and saw significant improvements in trust and engagement in research. Using the stakeholder engagement ladder, eight TSG members saw an increase in their engagement with clinical research by a minimum of two levels and maximum of five levels, and four TSG members remained at the same level from the convenings start to end. This demonstrates that intensive, leadership-based roles for community members in research can increase overall engagement and empowerment on short- to mid-term bases, dependent on consistent engagement and communication from study teams. The PEIRS yielded a significant change between time points 1 and 3, but no other pairwise comparisons reached Bonferroni-corrected statistical significance (Fig. 4). This suggests that a change in patient engagement over time may be subtle at shorter timescales, but with entrenched support and activity, changes in engagement become more visible. Although significant changes were not observed in measures of health empowerment, ceiling effects in the survey may have prevented sufficient variance to observe positive changes in this measure. Going beyond the charge and deliverables of a traditional CAB, TSG convening may be useful for academic health centers and industry groups alike to build sustainable community relationships that not only increase the likelihood of diverse recruitment and provide non-tokenized views of HMMs, but ensure community benefit is built into clinical trial design and research findings from conception. The TSG framework, implemented by organizations, may effectively anchor a broad array of research engagement and recruitment activities, including protocol-specific CBPR activities.

This approach to community engagement in clinical research requires relatively modest resources to facilitate ToC processes and adequately compensate TSGs for their expertise and time commitment. While not intended to be generalizable across all BIPOC communities or regions, this framework provides a method for community-based entrenchment that is highly localized and specificized dependent on the community chosen to engage, allowing researchers to improve relationships for a specific study or across study opportunities overall. Each study team looking to implement this practice should first determine the specific group they intend to work with and the scope of the problem, as advised in ToC convening guidelines, in order to adapt the work to the question at hand, and to improve generalizability. Implementing policies at institutional levels that require intersectional community involvement in research practice design is imperative to making this a sustainable model. Doing so will strengthen research practices overall and build bidirectional HMM relationships. These practices will improve research equity and outweigh initial costs of building said groups.

## Supporting information

Hemley et al. supplementary material 1 Hemley et al. supplementary material

Hemley et al. supplementary material 2 Hemley et al. supplementary material

## Supplementary material

The supplementary material for this article can be found at https://doi.org/10.1017/cts.2024.610.

## References

[1] Institute of Medicine. Transforming Clinical Research in the United States: Challenges and Opportunities: Workshop Summary. Washington, DC: The National Academies Press; 2010. doi: 10.17226/12900. 21210556

[2] Israel BA , Schulz AJ , Parker EA , Becker AB. Review of community-based research: assessing partnership approaches to improve public health. Ann Rev Public Health. 1998;19 **(** 1 **)**:173–202. doi: 10.1146/annurev.publhealth.19.1.173. 9611617

[3] Coakley M , Fadiran EO , Parrish LJ , Griffith RA , Weiss E , Carter C. Dialogues on diversifying clinical trials: successful strategies for engaging women and minorities in clinical trials. J Women’s Health. 2012;21 **(**7**)**:713–716. 10.1089/jwh.2012.3733 PMC3432572 22747427

[4] George S , Duran N , Norris K. A systematic review of barriers and facilitators to minority research participation among African Americans, Latinos, Asian Americans, and Pacific Islanders. Am J Public Health. 2014;104 **(** 2 **)**:e16–31. doi: 10.2105/ajph.2013.301706. PMC3935672 24328648

[5] Hatch J , Moss N , Saran A , Presley-Cantrell L , Mallory C. Community research: partnership in black communities. Am J Prev Med. 1993;9 **(** 6 **)**:27–31. doi: 10.1016/s0749-3797(18)30663-9. 8123284

[6] Clark LT , Watkins L , Pina IL , et al. Increasing diversity in clinical trials: overcoming critical barriers. Curr Probl Cardiol. 2019;44 **(** 5 **)**:148–172. doi: 10.1016/j.cpcardiol.2018.11.002. 30545650

[7] Breuer E , Lee L , De Silva M , Lund C. Using theory of change to design and evaluate public health interventions: a systematic review. Implement Sci. 2015;11 **(** 1 **)**:63. doi: 10.1186/s13012-016-0422-6. PMC4859947 27153985

[8] Healthy People 2010 Toolkit: A Field Guide to Health Planning. Public Health Foundation, under contract with the Office of Disease Prevention and Health Promotion, Office of Public Health and Science, U.S. Department of Health and Human Services; 2010: 46. https://www.phf.org/resourcestools/Pages/Healthy_People_2010_Toolkit.aspx

[9] Mainous AG , Smith DW , Geesey ME , Tilley BC. Development of a measure to assess patient trust in medical researchers. Ann Fam Med. 2006;4 **(** 3 **)**:247–252. doi: 10.1370/afm.541. 16735527 PMC1479445

[10] Hamilton CB , Hoens AM , McQuitty S , et al. Development and pre-testing of the Patient Engagement In Research Scale (PEIRS) to assess the quality of engagement from a patient perspective. PLoS One. 2018;13 **(** 11 **)**:e0206588. doi: 10.1371/journal.pone.0206588. 30383823 PMC6211727

[11] Israel BA , Checkoway B , Schulz A , Zimmerman M. Health education and community empowerment: conceptualizing and measuring perceptions of individual, organizational, and community control. Health Educ Q. 1994;21 **(** 2 **)**:149–170. doi: 10.1177/109019819402100203. 8021145

[12] Taplin DH , Clark H , Collins E , Colby DC. Theory of Change Technical Papers: a series of papers to support development of Theories of Change based on practice in the field. *ActKnowledge*, 2013. www.theoryofchange.org.

[13] Gienapp A , Reisman J , Stachowiak S. Getting started: A self-directed guide to outcome map development. ORS Impact, 2009. https://www.orsimpact.com/DirectoryAttachments/132018_25506_981_Getting-Started-Guide-FINAL-10-7-14.pdf.

[14] Brandt AM. Racism and research: the case of the Tuskegee syphilis study. Hastings Center Rep. 1978;6 **(** 6 **)**:21–29. doi: 10.2307/3561468. 721302

[15] Li V. *Thermo Fisher Scientific Inc. - An Extraordinary Event from More than 70 Years Ago That Led to a Lawsuit, Resulting in a Settlement*. Washington, DC**:** Food and Drug Law Institute (FDLI) Update, 2023: 21. https://www.fdli.org/2023/08/lacks-v-thermo-fisher-scientific-inc-an-extraordinary-event-from-more-than-70-years-ago/

[16] Chinekezi O, Andress L, Agonafer EP, Massick S , Piepenbrink S, Sutton KM , Alberti PM , de la Torre D , Guillot-Wright S , Lee M . From the national to the local: Issues of trust and a model for community-academic-engagement. Frontiers in Public Health, 2023;11. 10.3389/fpubh.2023.1068425. PMC10000727 36908463

