# Supplementary material for: A novel approach to research engagement: Developing a targeted theory of change with Black and African-American stakeholders
Source: J Clin Transl Sci. 2024 Oct 7;8(1):e143. doi: 10.1017/cts.2024.610 (PMC11523009; doi:10.1017/cts.2024.610)
Supplement: Hemley et al. supplementary material 1 — Hemley et al. supplementary material [file S2059866124006101sup001.pdf]

Stakeholder Convening to Develop a Targeted Theory of Change
Outcome Map Guidebook

How to use this Outcome Map:

Before research even happens, there needs to be a conversation with the community first. That is the intent of this Outcome Map – to start an ongoing, productive, and bidirectional relationship between communities identifying as Black, Indigenous and People of Color (BIPOC) and clinical research systems. This Outcome Map was not developed to recruit BIPOC persons for a single study or to ask for a collaboration with clear start and end dates. It is meant to start an ongoing process where both BIPOC communities in Boston, MA and clinical research systems have multiple entry points to reviewing whether the structures created together answer the following questions:

> A) Are we changing the proportion of BIPOC individuals who are participating in research?
>
> B) Are we building enough data to be used as a foundation, so that medical and scientific communities can tailor interventions for the high-risk populations we're discussing in the same way that privileged populations are?
>
> C) Are attitudes about research among BIPOC changing to become more accepting of research as a treatment option?

It is the hope that by using the Outcome Map as a guide for building this infrastructure that both BIPOC and researchers will benefit from expanding scientific knowledge of disease areas and that BIPOC communities will directly benefit from this research by engaging with clinical research on their own terms through a variety of entry points outside of formal recruitment. However, utilizing the Outcome Map shouldn't be the lead consideration when building towards this goal. It should be what research systems and communities alike use as a way to frame the results of the successful process of moving through the Theory of Change.

Community Ownership of Materials:

This Outcome Map was developed by 12 stakeholders from the Greater Boston Area who self-identify as being from BIPOC communities. As a result, this map should not yet be considered a final version as it has not received broader community input. As we start the process of gathering greater community feedback to finalize this map, this stakeholder group asks persons interacting with the Outcome Map to contact the group through Helen Hemley, MPA (hhemley@mgh.harvard.edu) to receive guidance on appropriately disseminating and replicating this work. It is fully the intent of the stakeholder group to allow other iterations of

this work to grow and flourish, but as this work is in the early stages, we must acknowledge the potential ease of having this Outcome Map used for unintended purposes and will act as gatekeepers for the immediate future.

Outcome Map Definitions

Defining Community: For the purpose of this Outcome Map, Community is defined as Black, Indigenous and People of Color (BIPOC) in the Greater Boston Area. The 12 stakeholders who developed this map self-identify as being from Black and African American communities but hope that this Outcome Map is generalizable for the broader BIPOC community.

Defining Systems: This Outcome Map defines systems as research infrastructures outside of individual researchers. This includes academic, government, non-profit and for-profit research institutions. All research systems that interact with the Greater Boston Area, whether they are physically located in the area or not and whether they have historically included BIPOC communities in their research or not are included in this definition.

Phases for Transformative Relationships via a Theory of Change

Contact Phase: A relationship between research systems and community where BIPOC members receive communication about clinical research and is given opportunities to provide feedback.

Partner Phase: A relationship between research systems and community where BIPOC members are engaged to better inform clinical research design, practice, and outreach through sustained interaction and collaboration.

Change Agent Phase: A relationship between research systems and community where BIPOC members are instrumental leaders in the process of providing feedback to and helping shape clinical research projects, programming, and infrastructure.

Systemic Barriers:

In order to build bidirectional relationships between BIPOC communities and research systems, these systems must first acknowledge the systemic barriers they have implemented and profited from at the cost to the health and well-being of BIPOC communities. While the stakeholder group has defined many of these barriers, listing them all is not the purpose of this Outcome Map. Each system must work, alone and with communities, to understand what systemic barriers they are implementing that are impeding BIPOC interaction with research. In addition to the barriers listed on the Outcome Map (Assumptions; Contextual factors; Historical factors), systemic barriers may include:

a. *Financial Barriers*
    i. The financial barriers for BIPOC communities to equitably be engaged, educated, and empowered to interact with research are many. To ensure equitable representation in research efforts, institutions must provide secure financial resources to both community and research groups that can help facilitate the process outlined in the Outcome Map. For example, as much as many research teams may like to have a multilingual staff to reach out to non-native English speakers, the reality is most teams cannot afford one. However, this should not be an issue for individual research teams to face alone and, instead, should have institutional resources to create equitable research entry points across all research teams. Research systems give the impression of caring about BIPOC communities when their financial resources are impacted. Because of this, it is paramount that research systems invest in BIPOC communities while, simultaneously, regulatory and funding mechanisms impose harsher financial restrictions on research systems that fail to carry out diverse and inclusive research.

b. *Active Discrimination*
    i. Barriers to BIPOC participation in research can include prior discriminatory treatment at a research facility or interaction with healthcare overall. Discriminatory treatment may look different to BIPOC communities than to research systems and the burden is on research systems to understand how interactions that are seemingly benign to researchers uphold communal trauma when interacting with health and research systems. As a result of this, it is hard to accurately capture the barriers of institutional culture and discrimination as a reason people don't want to be a part of research.

c. *Time Constraints*
    i. Research systems have created roadblocks that make research centering the needs of BIPOC communities difficult to implement, due to the perceived time and lack of funding for these areas. For example, research systems often lack personal connections within BIPOC communities and, rather than building long-term relationships, tend to put the burden of not participating in research on said communities. In order to reduce the amount of perceived time that individual researchers will have to take to establish relations relationships with community-based organizations, and to ensure that relationships are not broken when individual researchers leave, research systems must build and nurture these relationships. These will create avenues for those who do not have personal connections within the community to still begin to foster relationships with people from those communities. Institutions can be

intentional about investing in communities and community-based organizations to begin establishing relationships.

d. *Sustainability*

    i. Efforts at working with BIPOC communities around larger research engagement, empowerment, and education are often ad hoc and are not approached from the mindset of sustainability. For transformative change that reduces barriers, sustainable change and engagement must happen at a system level. Institutions will need to build long-term relationships and providing capital for community investments.
