# Supplementary material for: A novel approach to research engagement: Developing a targeted theory of change with Black and African-American stakeholders
Source: J Clin Transl Sci. 2024 Oct 7;8(1):e143. doi: 10.1017/cts.2024.610 (PMC11523009; doi:10.1017/cts.2024.610)
Supplement: Hemley et al. supplementary material 2 — Hemley et al. supplementary material [file S2059866124006101sup002.pdf]

Stakeholder Convening to Develop a Targeted Theory of Change

Theory of Change

Our Goal

The goal of this Stakeholder Group is to transform the relationship between clinical researchers, institutions, and Black, Indigenous and People of Color (BIPOC) in Boston, MA. We need to ensure that researchers and institutions are transparent, accountable, and willing to focus on long-term investments in BIPOC communities, rather than short-term personal benefits. The current structure of research often emphasizes an individual's career development and citations over community needs and values. Additionally, researchers and institutions must value the autonomy of each individual and respect their personal experience. Finally, we recognize that researchers and institutions have acknowledged historical barriers that still affect BIPOC communities. However, many modern barriers (cultural in concordance, discrimination, income, education, technology, communication, healthcare access) that have a greater impact on inequity and mistrust have not been confronted. To advance equity in research engagement, recruitment, and retention, researchers will need to first dismantle this system and replace it with community-centric models of research engagement.

To accomplish this, researchers and institutions are required to meaningfully invest in and build relationships with underrepresented communities. The first time they interact with BIPOC individuals cannot be when minority participants are needed for a study. These investments may take the form of education (i.e. ensuring that everyone knows their rights as a research participant), supporting health initiatives that are chosen and designed by community experts, and/or investing in research infrastructure led and created by the community. It is vital that future research engagement is designed by and for BIPOC communities to ensure that all individuals, not just those engaged in research, have access, and receive tangible benefits. Finally, once bidirectional relationships are built with the community and systemic barriers that uphold racism within research engagement have been accounted for internally by researchers and institutions, BIPOC communities across Boston must be given the tools to advocate for themselves. Researchers and institutions must empower BIPOC communities to become involved in every stage of the research process, from proposal to publication. We believe these efforts will begin to bridge the gap between researchers, institutions, and our community, creating equitable opportunities for all of us to benefit from research.

Objectives for Bidirectional Community Engagement

Our Theory of Change model addresses barriers to research via three channels: Empowerment, Engagement, and Education. We define empowerment here as a patient's ability to freely ask questions, feel fully confident to advocate on their own behalf in research matters, understand how a study will affect their health and/or insurance, and know what is required of them and/or their community if they participate. We define engagement as any interaction with research outside of recruitment, such as participating in research design, attending informational sessions and giving feedback, or having a chance to learn more about research

outside of clinical settings. The burden of effort in engagement activities should fall on researchers and institutions. They must be willing to travel to the community to ensure these opportunities are accessible. Additionally, they must partner with people from the community who understand the dynamics of race relations in Boston. Finally, we define education in three parts: First, as the training of research staff and institutions in current barriers and health concerns facing BIPOC communities in Boston and eliminating discrimination in the research process. Second, as the direct education of the community by research staff in research and its benefits for present and future generations. Third, as institutions building long-term relationships with the community and providing capital to compensate community members for their time spent on these partnerships.

Clinical researchers often refer to historical atrocities, such as the Tuskegee Syphilis Study or Henrietta Lacks, as enduring factors in medical and research mistrust. However, within BIPOC communities, these are not seen as prominent in advancing racial health disparities as modern systemic barriers built into clinical research and healthcare. Within the current culture, researchers can be reluctant to fully engage with BIPOC participants or take their concerns seriously. When research systems view clinical research engagement holistically and adopt a collaborative mindset, each patient can then participate equally in the decision-making process, providing opportunities for the community, clinical research systems, and BIPOC researchers to equitize research engagement:

- *Community relationships*: Investment in community expertise and long-standing relationship building should create avenues for community members to provide forthright feedback on their research experiences and design improved channels for community engagement and empowerment. With these changes, potential BIPOC communities may feel comfortable speaking out when transgressions occur.
- *Research systems improvement*: By investing in community expertise to enhance research education and empowerment models, researchers can build bidirectional relationships with community leaders and participants alike, reducing the need to make assumptions about reasons for lack of study representation. This feedback and participants' outcomes should be tracked and incentivized, so researchers are rewarded when they provide measurable benefits to our community and traditional metrics for success within research systems are balanced with community values.
- *Representative research retainment*: BIPOC individuals who are current or aspiring researchers must be empowered to continue their research within Boston. The Boston clinical research system has not been able to retain BIPOC researchers due to reports of isolation and systemic misunderstanding of the effects of generational social determinants of health and racism. However, they are not addressing the mechanisms that are pushing BIPOC researchers out in the first place. Institutions must improve pay, identify all factors that are causing researchers to leave, and create supports for researchers to be retained within Boston health and research systems. Additionally, institutions should invest locally in students interested in medical careers, to ensure that our community is represented. Finally, institutions need to address why many diverse applicants are not hired in Boston, despite major diversity hiring initiatives.

Once BIPOC communities in Boston are empowered by and educated about clinical research infrastructure, successfully engagement that leads to equity in clinical research can begin. However, researchers need to view community members as active and equal partners in their work, not a means to an end. Methods for initiating this process may include:

- Creating broader educational marketing campaigns in which researchers explain how BIPOC communities in Boston will be directly impacted, beyond the scope of an individual study.
- Improve Institutional Review Board (IRB) processes by considering administrative barriers that impede engagement, such as overly complex consent forms or red tape that slows down researcher's progress in addressing community's needs.
- Institutional investment in community-owned IRBs and clear guidelines on inclusive representation. These IRBs should not be limited to current patients at an institution. Rather, they should include those who live and are most directly affected by changes in the community.
- Continued engagement with youth populations, as positive research experiences at a young age may help to break the cycle of mistrust and increase pipelines for research careers.
- Ensure that institutions and researchers have the right information and training to serve BIPOC communities. For example, it's often assumed that monetary payment is the only incentive needed, particularly for low-income individuals. Instead, institutions should work with community members to evaluate what potential participants may value, such as education and ease of access around community health concerns.
